# A new transgenic rice line exhibiting enhanced ferric iron reduction and phytosiderophore production confers tolerance to low iron availability in calcareous soil

**DOI:** 10.1371/journal.pone.0173441

**Published:** 2017-03-09

**Authors:** Hiroshi Masuda, Erika Shimochi, Tatsuro Hamada, Takeshi Senoura, Takanori Kobayashi, May Sann Aung, Yasuhiro Ishimaru, Yuko Ogo, Hiromi Nakanishi, Naoko K. Nishizawa

**Affiliations:** 1 Research Institute for Bioresources and Biotechnology, Ishikawa Prefectural University, Nonoichi, Ishikawa, Japan; 2 Graduate School of Science, Tohoku University, Aoba, Sendai, Miyagi, Japan; 3 Functional Plant Research Unit, National Institute of Agrobiological Sciences, Tsukuba, Ibaraki, Japan; 4 Graduate School of Agricultural and Life Sciences, The University of Tokyo, Bunkyo, Tokyo, Japan; RIKEN Center for Sustainable Resource Science, JAPAN

## Abstract

Iron (Fe) deficiency is a critical agricultural problem, especially in calcareous soil, which is distributed worldwide. Rice plants take up Fe(II) from soil through a OsIRT1 transporter (Strategy I-related system) and also take up Fe(III) via a phytosiderophore-based system (Strategy II system). However, rice plants are susceptible to low-Fe conditions because they have low Fe(III) reduction activity and low-level phytosiderophore secretion. Previously, we produced transgenic rice plants expressing a mutationally reconstructed yeast ferric chelate reductase, *refre1/372*, under the control of the *OsIRT1* promoter. This transgenic rice line exhibited higher Fe(III) chelate reductase activity and tolerance to Fe deficiency. In addition, we produced transgenic rice overexpressing the Fe deficiency-inducible transcription factor, OsIRO2, which regulates the expression of various genes involved in the strategy II Fe(III) uptake system, including *OsNAS1*, *OsNAAT1*, *OsDMAS1*, *OsYSL15*, and *TOM1*. This transgenic rice exhibited improved phytosiderophore secretion ability and tolerance to Fe deficiency. In the present research, transgenic rice plants that possess both the *OsIRT1* promoter-*refre1/372* and the *35S* promoter-*OsIRO2* (RI lines) were produced to enhance both Strategy I Fe(II) reductase ability and Strategy II phytosiderophore productivity. RI lines exhibited enhanced tolerance to Fe-deficient conditions at the early and middle-late stages of growth in calcareous soil, compared to both the non-transgenic line and lines harboring either *OsIRT1* promoter-*refre1/372* or *35S* promoter-*OsIRO2* alone. RI lines also exhibited a 9-fold higher yield than the non-transgenic line. Moreover, we successfully produced Fe-deficiency-tolerant Tachisugata rice, which is a high-biomass variety used as fodder. Collectively, our results demonstrate that combined enhancement of two Fe uptake systems in rice is highly effective in conferring tolerance to low Fe availability in calcareous soil.

## Introduction

Iron (Fe) deficiency is a widespread agricultural problem that is common in calcareous soils, which cover more than 30% of the earth’s surface. Although there are abundant minerals in soil, Fe is sparingly soluble under aerobic conditions, in particular at high pH in calcareous soil [1]. Consequently, plants often exhibit Fe-deficiency symptoms, such as chlorosis, leading to reduced crop yield and quality. Thus, Fe is a key determinant of biomass production and of plant product quality [2]. Development of crops tolerant to low Fe availability is important to meet the increased demand for food caused by rapidly increasing populations. Moreover, cultivation of high-biomass crops tolerant to low Fe availability in calcareous soil would reduce the carbon dioxide concentration in the atmosphere and, consequently, ameliorate global warming.

Higher plants use two major Fe uptake strategies—Strategy I and Strategy II [3]. Under conditions of low Fe availability, non-graminaceous plants use the Strategy I system, which involves induction of two major processes. First, Fe(III)-chelate reductase (FRO) is induced in roots and reduces Fe(III)-chelate to Fe^2+^ [4], which is assumed to be the rate-limiting step for Fe acquisition from soil [5]. Next, the Fe^2+^ is absorbed via the Fe-regulated transporter (IRT) [6], which is the major Fe^2+^ transporter in plant roots.

Although rice is a graminaceous plant, rice also possesses a direct Fe^2+^ uptake system mediated by the ferrous transporter OsIRT1. This is a part of the Strategy I system [7–8]. In paddy fields (where rice plants are normally grown), Fe^2+^ is abundant because of the low redox potential, which is assumed to be the reason why rice plants possess Fe^2+^ uptake systems [7–8]. However, rice plants have a low Fe(III)-chelate-reductase activity [8], and thus lack a complete Strategy I system and show limited Fe uptake efficiency.

The Strategy II mechanism is specific to graminaceous plants, which synthesize and secrete mugineic acid family phytosiderophores (MAs) into soil; these compounds chelate sparingly soluble Fe(III) [9]. Secretion of MAs from roots to the rhizosphere is mediated by the “transporter of mugineic acid 1” (TOM1:Nozoye *et al*. [10]). The resulting Fe(III)–MAs complexes are solubilized and absorbed into the root by the Fe(III)-MAs transporters termed Yellow Stripe 1 (YS1) and Yellow Stripe-Like (YSL) of the plasma membrane, such as maize ZmYS1 [11], rice OsYSL15 [12–13], and barley HvYS1 [14]. The biosynthetic pathway of MAs from methionine, via nicotianamine (NA) as an intermediate, has been elucidated [15–23]. Nicotianamine synthase (NAS) catalyzes *S*-adenosyl-L-methionine to form NA [17], which is then converted into the 3″-oxo intermediate via transfer of an amino group by nicotianamine aminotransferase (NAAT) [19]. Subsequently, deoxymugineic acid synthase (DMAS) produces 2′-deoxymugineic acid (DMA) from the 3″-oxo intermediate [23]. All the MAs share the biosynthetic pathway from *S*-adenosyl-L-methionine to DMA, which is then converted to other MAs in several graminaceous plants via deoxygenases, such as IDS3 and IDS2 [20–21]. Rice plants also synthesize and secrete MAs under conditions of Fe deficiency. Nozoye *et al*. [24] demonstrated that OsNAS2 was localized to vesicles in Fe-deficient roots; transport of these vesicles is a crucial step in NA synthesis by rice, in turn leading to DMA synthesis and secretion. However, rice secretes lower levels of MAs than do other graminaceous crops, such as barley [25]. This is one of the reasons why rice, among the graminaceous plants tested to date, is the most susceptible to Fe deficiency.

To produce Fe-deficiency-tolerant rice, the first approach used was to enhance biosynthesis of MAs in the plant. Takahashi *et al*. [26] produced transgenic rice carrying a barley genome fragment containing the *HvNAAT-A* and *HvNAAT-B* genes, and demonstrated that the transgenic rice was tolerant to Fe deficiency in a calcareous soil. Suzuki *et al*. [27] carried out field experiments with transgenic rice lines harboring the barley genomic fragment of either *HvNAS1* or *HvNAS1* plus *HvNAAT-A* and *HvNAAT-B*, or *IDS3*; all rice lines were tolerant to Fe deficiency. These results support the notion that rice with enhanced phytosiderophore productivity exhibits tolerance to Fe deficiency.

The second approach used was to enhance the Fe(III)-chelate reductase activity in rice. To this end, Oki *et al*. [28] artificially reconstructed and mutagenized the yeast Fe(III)-chelate reductase gene *FRE1*, to generate *refre1/372*, whose encoding protein exhibits enhanced enzymatic activity at high pH, to facilitate growth in calcareous soils. Ishimaru *et al*. [29] introduced *refre1/372* into rice plants under the control of the *OsIRT1* promoter, which drives gene expression predominantly in the root epidermis in response to Fe deficiency [8]. Such transgenic rice plants exhibited higher Fe(III)-chelate reductase activity and enhanced tolerance to low Fe availability in calcareous soils [29]. The grain yield was 7.9-fold higher than that of non-transformants (NT).

The third approach used was to enhance expression of transcription factors that control Fe homeostasis-related genes in rice. Ogo *et al*. [30] identified an Fe deficiency-inducible basic helix–loop–helix (bHLH) transcription factor, OsIRO2, in rice. OsIRO2 is responsible for regulation of the key genes involved in Strategy II-related Fe uptake; e.g., *OsNAS1*, *OsNAS2*, *OsNAAT1*, *OsDMAS1*, *TOM1*, and *OsYSL15* [31–32]. Ogo et al. [31] introduced *OsIRO2* under the control of the constitutive cauliflower mosaic virus *35S* promoter (*35S* promoter) into rice. *OsIRO2*-overexpressing rice secreted a greater quantity of DMA than did NT plants and exhibited enhanced tolerance to Fe deficiency in calcareous soils [31–32]. Based on these results, we hypothesize that transgenic rice plants with combined enhancement of *refre1/372* (second approach) and *OsIRO2* (third approach) would be more tolerant to low Fe availability, because refre1/372 and OsIRO2 enhance the activities of two different Fe acquisition systems, Strategy I and Strategy II, respectively.

In the present study, firstly, we produced transgenic rice lines using the Tsukinohikari cultivar with Fe deficiency-inducible expression of *refre1/372* under the control of the *OsIRT1* promoter, with constitutive overexpression of *OsIRO2*, and evaluated their tolerance to low Fe availability in calcareous soil. Introduction of both *refre1/372* and *OsIRO2* was more effective than single introduction of either gene under water-submerged conditions. Additionally, Ohta *et al*. [33] developed a high-biomass rice variety, Tachisugata, the entire silage of which is used as fodder. Thus secondly, we also produced Fe-deficiency-tolerant Tachisugata rice by introducing the *refre1/372* and *OsIRO2* genes.

## Materials and methods

### Production of refre1/372-OxOsIRO2 lines (RI lines)

The plasmid pIG121Hm containing the *35S* promoter-*OsIRO2* ORF [31] was used as a PCR template. The *35S* promoter-*OsIRO2* ORF fragment lacking a *Hin*dIII site at the 3′ end was amplified using the primers 5′-TAT AAG CTT GCA TGC CTG CAG GTC-3′ and 5′-TTA GAG TTT TGC TTT GTT CCT GAC G-3′. The amplified fragment was ligated to the nopaline synthase terminator (AF485783) fragment, excised from pE7133-*GUS* [34] by *Ecl*136II and *Eco*RI, using T4 DNA ligase (TaKaRa, Japan) and subsequently amplified again, using the primers 5′-TAT AAG CTT GCA TGC CTG CAG GTC-3′ and 5′-ATA AAG CTT CCG ATC TAG TAA CAT AGA TG -3′, to append an *Hin*dIII site to the 3′ end of the nopaline synthase terminator. This amplified fragment was subcloned into the pTA2 vector (TOYOBO, Japan), verified by DNA sequencing, and finally inserted into pIG121Hm containing the *OsIRT1* promoter-*refre1/372* [29] at the *Hin*dIII site to construct pIG121Hm-Refre1/372-OxOsIRO2. *Agrobacterium tumefaciens* strain EHA105 transformed with pIG121Hm-Refre1/372-OxOsIRO2 was used to transform two rice cultivars (*Oryza sativa* L. cv. Tsukinohikari and Tachisugata).

Transformation of the rice cultivar Tsukinohikari was performed using the method outlined by Sallaud *et al*. [35] and Terada *et al*. [36]. Most transgenic procedures for transformation of the Tachisugata rice cultivar are based on the same method, with the following exceptions. Calli were cultivated at 30°C throughout all rice transformation steps except co-cultivation with Agrobacterium at 23°C. For regeneration, Murashige and Skoog (MS) salts and vitamins (PhytoTechnology Laboratories, Kansas, USA), 30 g/L sorbitol (Wako, Japan), 30 g/L maltose (Wako, Japan), 2 g/L casamino acids (Nihon Pharmaceutical, Japan), 2 mg/L kinetin (Wako, Japan), 2 μg/L NAA (Wako, Japan), 10 mg/L hygromycin B (Wako, Japan), and 5 g/L gelrite (San-Ei Gen F.F.I., Japan) formed the regeneration medium. Hygromycin B at 15 and 10 mg/L was used for selection, and regeneration and rooting, respectively.

After confirmation of gene insertion into regenerated plants by genomic PCR as described below, the transformants (RI lines) were cultivated in a greenhouse at 28°C under natural light until mature T_1_ seeds were obtained.

### Detection of inserted genes of transgenic lines by genomic PCR

Genomic DNA was extracted from the leaves of Tsukinohikari-RI T_0_ transgenic rice plants using the method of Thomson and Henry [37], and introduction of *OsIRO2* and *refre1/372* was confirmed using KOD FX (TOYOBO, Japan). The primers used for genomic PCR of *OsIRO2* were 5′-ATG GAG CAG CTG TTC GTC GAC G-3′ and 5′-TTA GAG TTT TGC TTT GTT CCT GAC G-3′. The primers used for genomic PCR of *refre1/372* were 5′-TAA CAA GAC TCT GGA CTC CGC TTT G-3′ and 5′-TAG AAC CAG GCT GAT TTT GGT GAA A-3′. The pIG121Hm-Refre1/372-OxOsIRO2 plasmid was used as a PCR template for confirmation of band size.

### RNA preparation and quantitative RT-PCR

NT and T_1_ seeds of Tsukinohikari-RI transgenic rice were germinated for 13 days on MS medium (sucrose 30 g/L, NH_4_NO_3_ 1.65 g/L, KNO_3_ 1.9 g/L, CaCl_2_•2H_2_O 440 mg/L, MgSO_4_•7H_2_O 370 mg/L, KH_2_PO_4_ 170 mg/L, Fe(III)-EDTA 42.1 mg/L, H_3_BO_3_ 6.2 mg/L, MnSO_4_•4H_2_O 22.3 mg/L, ZnSO_4_•7H_2_O, 8.6 mg/L, KI 0.83 mg/L, Na_2_MoO_4_•2H_2_O 250 ng/L, CuSO_4_•5H_2_O 25 ng/L, CoCl_2_•6H_2_O 25 ng/L, thiamine-HCl 100 ng/L, nicotinic acid 500 ng/L, pyridoxine-HCl 500 ng/L, glycine 2 mg/L, *myo*-inositol 100 mg/L, and agar 8 g/L, pH 5.8) with hygromycin (50 mg/L, for transgenic plants) or without hygromycin (for NT plants) at 28°C under 24 h-light conditions. Then, plantlets were acclimated for 3 days and transferred to a 15 L plastic container containing a hydroponic culture solution of the following composition: K_2_SO_4_ 122 mg/L, KCl 7.5 mg/L, KH_2_PO_4_ 14 mg/L, Ca(NO_3_)_2_•4H_2_O 472 mg/L, MgSO_4_•7H_2_O 123 mg/L, H_3_BO_3_ 0.62 mg/L, MnSO_4_•5H_2_O 0.12 mg/L, ZnSO_4_•7H_2_O 0.14 mg/L, CuSO_4_•5H_2_O 0.05 mg/L, (NH_4_)_6_Mo_7_O_24_ 0.012 mg/L and Fe(III)-EDTA 42 mg/L. The nutrient solution was adjusted to pH 5.5 with 1 M HCl every 2 days and renewed weekly. After 2 weeks, plants were transferred to a hydroponic culture solution without Fe(III)-EDTA and grown for a further 7 days for exposure to Fe-deficiency. Total RNA was extracted from roots using an RNeasy Plant Mini Kit (QIAGEN, Germany). First-strand cDNA was synthesized from extracted total RNA using a Rever Tra Ace qPCR RT Kit (TOYOBO, Japan). Quantitative RT-PCR was performed using a 7300 Real-Time PCR system (Applied Biosystems, California, USA) and GoTaq^®^ qPCR Master Mix (Promega, Wisconsin, USA). The primers used were as follows: 5′-GGC ATG GCT CCC ATC GT-3′ and 5′-AAC AAG CTG ACC TGA ACC ATG A-3′ (*OsIRO2*), and 5′-TCA CGC CGT GCT GAC TTG-3′ and 5′-TCC GGA TAC CGA AAA GGT ACA-3′ (*refre1/372*). Transcript levels were normalized to the expression levels of alpha-*Tubulin*, as determined using the primers 5′-GCA ACT CTC TGT TGC CGA GAT-3′ and 5′-GTC GCA CTT GGC CAT CAT G-3′. The sizes of the amplified fragments were confirmed by agarose gel electrophoresis.

T_3_ seeds of Tsukinohikari-RI line Nos. 21 and 22, seeds of Tsukinohikari-Refre1 line No. 7 [29], seeds of Tsukinohikari-IRO2 line No. 2 (Ogo *et al*. [31], OX2), and non-transgenic (NT) Tsukinohikari seeds were also germinated and cultivated in hydroponic culture solution as described above for 10 days under Fe-sufficient conditions, and then grown in Fe-deficient hydroponic culture without Fe(III)-EDTA for 1 day. After RNA extraction and cDNA synthesis as described above, quantitative RT-PCR was performed using StepOnePlus^™^ Real-Time PCR System (Life technologies, Tokyo, Japan) using primers used in [10] for *OsTOM1*, those in [12] for *OsYSL15* (5′-ACT GGT ACC CTG CAA ACA TAC-3′ and 5′-GCA ATG ATG CTT AGC AAG AAG-3′), and those in [38] for *OsNAS1*, *OsNAS2*, *OsNAAT1* and *OsDMAS1*.

### Root Fe(III)-chelate reductase activity assay

Tsukinohikari-RI plants were germinated on MS medium. Two weeks later, plantlets were cultivated in hydroponic culture solution as described above for 10 days under Fe-sufficient conditions, and then cultured in Fe-deficient culture solution without any form of Fe. Root Fe(III)-chelate reductase activities in whole intact root systems were determined as described previously [29] at 3, 5, 7, and 10 days after onset of Fe-deficient cultivation. Roots were rinsed with water and submerged in 40 mL of assay solution (0.2 mM CaSO_4_, 5 mM 2-[4-(2-hydroxyethyl)-1-piperazinyl] ethanesulfonic acid at pH 5.5, 0.1 mM Fe(III)-EDTA, and 0.2 mM bathophenanthroline disulfonic acid disodium salt) (Wako, Japan). After incubation for 1 h at 25°C, an aliquot of the assay solution was collected, and the absorbance of the solution at 535 nm was determined using a UV-2450 spectrophotometer (SHIMADZU, Japan). The amount of Fe^2+^ produced was calculated from a standard curve prepared using standard solutions of Fe^2+^.

### Growth test of RI lines on calcareous soil and yield analysis

T_2_ seeds of Tsukinohikari-RI line Nos. 21 and 22, T_2_ seeds of Tsukinohikari-Refre1 line No. 7 [29], T_2_ seeds of Tsukinohikari-IRO2 line No. 2 (Ogo *et al*. [31], OX2), and non-transgenic (NT) Tsukinohikari seeds were used for growth testing in calcareous soil. Seeds were germinated on MS medium with hygromycin 50 mg/L (for transgenic seeds) or without hygromycin (for NT seeds) for 20 days. Then, each plantlet was acclimated for 3 days and then transferred to a pot containing 1 kg calcareous soil obtained from Takaoka City, Toyama, Japan (Nihonkai Kougyou, Japan; pH 8.9, soluble CaO: 39.6%, Fe_2_O_3_: 1.7%); the soil was autoclaved just before transplanting to prevent pest contamination, then supplied with the slow-release fertilizers Eco long total 70 and Eco long total 140 containing N:P:K = 13:11:13, Fe 0.20% as EDTA-Na-Fe(III), Cu 0.050%, Zn 0.015%, and Mo 0.020% (JCAM AGRI, Japan) 3.5 g/pot. A netting sheet was placed on the base of each pot to prevent the roots from growing outside the pots. The plants were grown constantly submerged in water in a greenhouse at 28°C under natural light conditions. Shoot height and the SPAD value of the newest leaf were measured once every three days using a SPAD-502 chlorophyll meter (Konika Minolta, Japan) until 58 days after transplanting (DAT), thus to the panicle initiation stage. After seed maturation, water submergence was continued until 151 DAT. Tiller numbers were counted at 151 DAT. The water supply then ceased for 28 days, then plants were harvested on 179 DAT. Plant heights, and the dry weights of straws and panicles, were measured on the day of harvesting. The number of grains per panicle, the number of panicles per plant, and the total number of grains were measured. Fully matured grains (specific gravity >1.06) were selected by salty water. The filled grain rate, the 1,000-grain weight, and total grain weight of fully matured grains were calculated.

T_1_ seeds of Tachisugata RI lines were germinated on MS medium with hygromycin 30 mg/L without Fe, and incubated at 13°C for 1 week. Next, the seeds were incubated at 32°C for 1 day. After germination, each plantlet was grown in 400 mL of calcareous soil in a pot. Ion-exchanged water (50 mL) and half-concentration Fe-deficient hydroponic culture solution (50 mL; pH 8.0) described above were supplied to each pot on alternating days.

### Measurement of metal content

Plants grown in calcareous soil were dried for 2 days at 60°C. Metal concentration analysis was carried out according to the method of Masuda *et al*. [39]. Ten brown-rice grains were digested with 1 mL HNO_3_ and 1 mL H_2_O_2_ for 20 min at 200°C in a Mars Xpress oven (CEM, NC, USA). After digestion, the samples were collected in a tube and the volume made up to 5 mL with 3 mL 0.1 M HCl. Next, the metal content was measured using an ICPS-8100 (SHIMADZU, Kyoto, Japan) at wavelengths of 238.204 nm (Fe), 257.610 nm (Mn), 202.551 nm (Zn), and 324.754 nm (Cu). Straw was digested with 2 mL of HNO_3_ and 2 mL of H_2_O_2,_ and the metal contents measured using the method described above.

### Statistical analysis

For the Fe(III)-chelate reductase activity assay, we grew three plants of each variety (NT, IRO2, Refre1, and RI lines 21 and 22) as biological replicates (n = 3) and for calcareous cultivation, we grew four plants of each variety (n = 4). Analysis of variance (ANOVA) via Student’s *t*-test was used to examine experimental data from both the Fe(III)-chelate reductase activity assay and calcareous soil cultivation, such as plant height, tiller number, weight of straw, weight of panicle yield index, and Fe content per plant; we employed JMP9 software (SAS Institute, Cary, NC, U.S.A.) to this end and *p* < 0.05 was considered statistically significant.

## Results

### Selection of transgenic lines with higher expression levels of *OsIRO2* and *refre1/372* in roots under Fe-deficiency conditions

**Fig 1:**
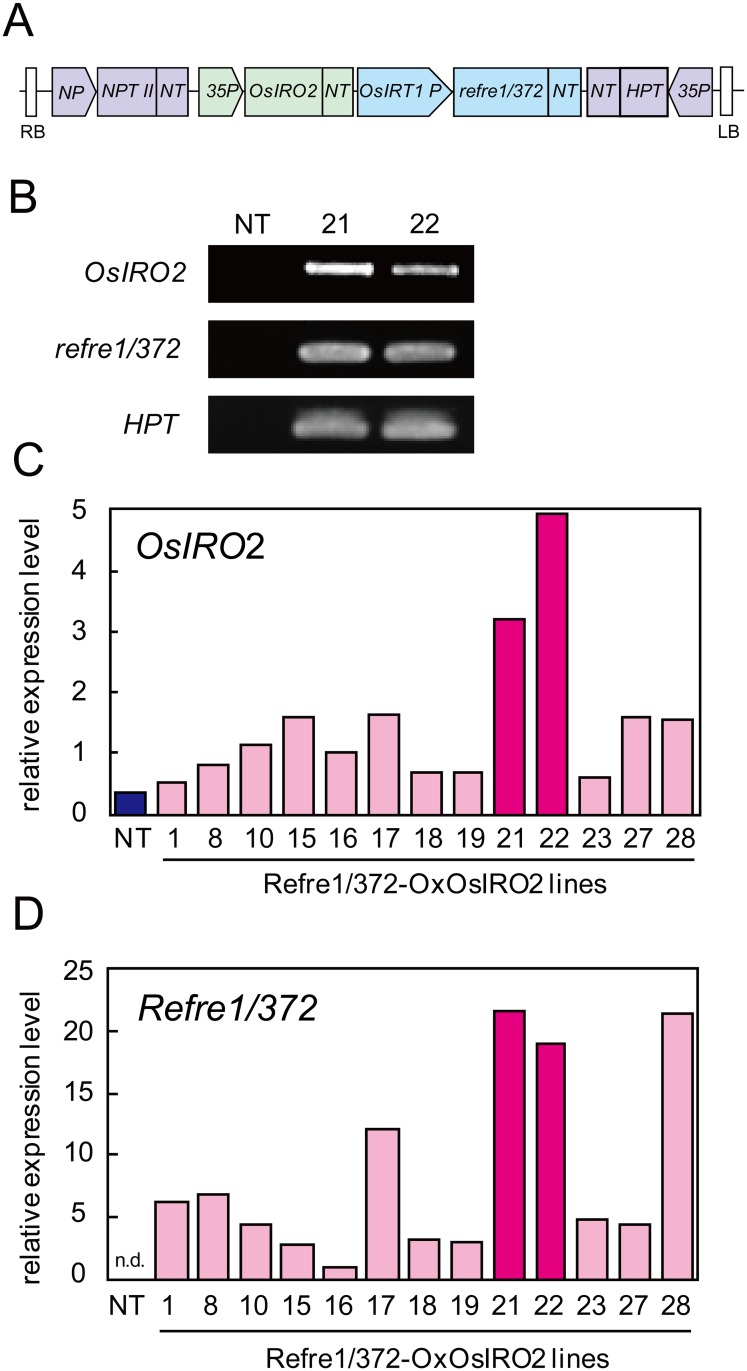
Production of RI rice. (A) The gene cassette introduced into rice to produce RI lines. LB, left border; RB, right border; *NP*, nopaline synthase promoter; *NPT II*, neomycin phosphotransferase II; *NT*, nopaline synthase terminator; *35P*, cauliflower mosaic virus 35S promoter; *OsIRO2*, ORF region of the Fe deficiency-inducible bHLH transcription factor gene *OsIRO2* [31]; *OsIRT1 P*, 0.8-kb 5' upstream region of the *OsIRT1* gene [8]; *refre1/372*, the mutationally reconstructed ferric-chelate reductase gene *refre1/372* from yeast [28]. *HPT*, hygromycin phosphotransferase. The plasmid backbone was that of the pIG121Hm binary vector [40]. (B) Confirmation of gene insertion in RI lines by genomic PCR. NT, non-transgenic line; 21 and 22, RI lines 21 and 22. Gene insertions in the other RI lines and the pIG121Hm-*refre1/372-OxOsIRO2* plasmid template were confirmed (the band sizes were identical). (C, D) *OsIRO2* and *refre1/372* expression levels as determined by quantitative RT-PCR. (C) *OsIRO2* expression. (D) *refre1/372* expression. T_1_ transgenic rice and NT lines were grown in hydroponic culture solution for 2 weeks and transferred to Fe-deficient culture solution for 7 days. Total RNA was extracted from roots of rice plants and gene expression levels were analyzed. n.d., not detected.

A construct harboring the *35S* promoter-*OsIRO2* and the *OsIRT1* promoter-*refre1/372* was introduced into rice plants by *Agrobacterium*-mediated transformation (Fig 1A). We used genomic PCR to confirm the introduction of *OsIRO2* and *refre1/372* into 28 regenerated plant lines. Among these, 24 lines were found to include both inserted genes and we termed these lines ‘RI lines’. As representatives, the genomic PCR results of line numbers 21 and 22 compared with NT are shown in Fig 1B. T_1_ plants were cultivated under Fe-deficient conditions, and *OsIRO2* and *refre1/372* expression levels in roots were analyzed by quantitative RT-PCR (Fig 1C and 1D). The expression levels of *OsIRO2* in the RI lines were higher than those in the NT line (Fig 1C). *refre1/372* expression was detected in RI lines but not in the NT line (Fig 1D). The expression levels of *OsIRO2* and *refre1/372* were higher in RI lines 21 and 22 than in all or most of the other transgenic lines and the NT line. Therefore, we selected RI lines 21 and 22 to obtain T_2_ and T_3_ seeds, which were then used for further analysis.

We confirmed the expression levels of *OsIRO2*, *refre1/372* and representative genes regulated by OsIRO2 such as *OsNAS1*, *OsNAS2*, *OsNAAT1*, *OsDMAS1*, *OsYSL15* and *TOM1* in hydroponically grown roots after 1-day Fe deficiency cultivation (S1 Fig). Among these genes, *OsNAS1*, *OsNAS2*, *OsYSL15* and *TOM1* showed higher expression level in IRO2 line compared to NT, and in RI lines 21 and 22 compared to Refre1 line. *OsNAAT1* and *OsDMAS1* were expressed at higher levels in RI lines 21 and 22 compared to Refre1 line.

### Fe-deficient hydroponic culture and root Fe(III)-chelate reductase activity

**Fig 2:**
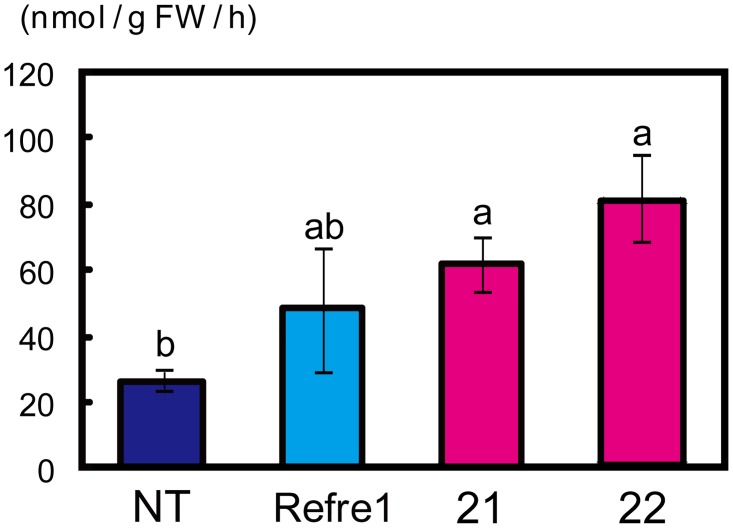
Assay of Fe(III)-chelate reductase activity. The non-transgenic line (NT), transgenic rice lines harboring *OsIRT1* promoter-*refre1/372*’ (Refre1), and RI lines 21 and 22 were grown in hydroponic culture solution with Fe for 10 days and then transferred to hydroponic culture solution without Fe. Fe(III)-chelate-reductase activity of roots growing under Fe-deficiency conditions for 3 days were measured (means ± standard error, n = 3). Values with different letters were significantly different by Student’s *t*-test (*p* < 0.05).

T_3_ plants of RI lines 21 and 22 and the NT line were grown for 10 days in Fe-sufficient hydroponic culture solution and then transferred to Fe-deficient hydroponic culture solution. Relative chlorophyll contents (measured as SPAD values) of leaves and Fe(III)-chelate reductase activities were determined. T_3_ plants of the *OsIRO2*-overexpressing transgenic rice (IRO2 line, [31]) and T_3_ plants of transgenic rice expressing the *OsIRT1* promoter-*refre1/372* (Refre1 line, [29]) were also grown and analyzed. The SPAD values of IRO2 and RI lines 21 and 22 were higher than those of the Refre1 and NT lines at 3–10 DAT to Fe-deficient hydroponic culture solution (S2A Fig). At 3 DAT, Fe(III)-chelate reductase activities in RI lines 21 and 22 were 2.8- and 3.1-fold that of the NT line, respectively, and were comparable to the Refre1 line (Fig 2). However, the activities of RI lines 21 and 22 decreased to become lower than that of the Refre1 line at 5 and 7 DAT (S2B Fig).

### Tolerance of RI lines to Fe deficiency in calcareous soil

**Fig 3:**
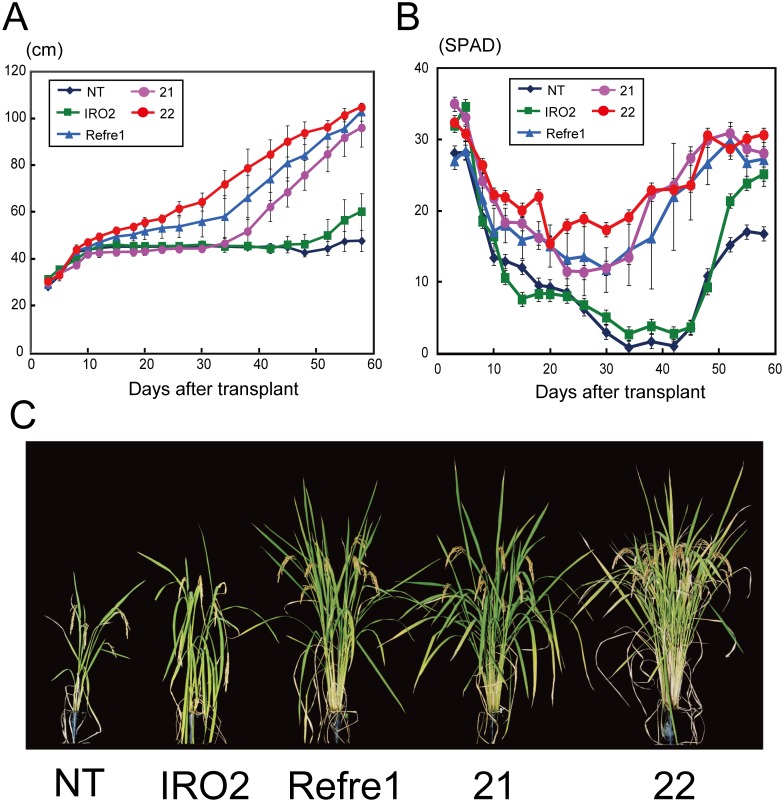
Growth test of Tsukinohikari-RI rice lines in calcareous soil. (A) Plant heights after transplantation to calcareous soil. (B) SPAD values (chlorophyll content) of the newest leaves after transplantation to calcareous soil. Means ± standard error, n = 4. (C) Photograph of plants at 144 DAT. NT, non-transgenic line; IRO2, *OsIRO2*-overexpressing transgenic rice; Refre1, *OsIRT1* promoter-*refre1/372*’ transgenic rice; 21 and 22, RI transgenic rice lines 21 and 22.

T_3_ plants of the IRO2 and Refre1 lines, T_2_ plants of RI lines 21 and 22, and the NT line were grown in calcareous soil (pH = 8.9) under water submerged conditions (S3 Fig). Four plants were cultivated per line. Plant heights and SPAD values of the new leaves were measured (Fig 3A and 3B). The plant growth of RI line 22 and the Refre1 line increased continuously (Fig 3A), while those of the NT and IRO2 lines and RI line 21 did not increase after 10 DAT. The plant height of RI line 21 increased again from 30 DAT, as it gradually recovered from chlorosis. The SPAD values of all plants decreased after transplantation to calcareous soil (Fig 3B). During the first week after transplantation, RI line 21, RI line 22, and the IRO2 line showed markedly higher SPAD values and higher tolerance to low Fe availability compared to the NT and Refre1 lines. After 1 week, the SPAD value of the IRO2 line decreased markedly, whereas those of RI lines 21 and 22, and Refre1 lines decreased to a lesser extent, compared to the NT and IRO2 lines, and then recovered gradually beginning at 30 DAT (Fig 3B). The SPAD value of RI line 22 was higher than those of the other lines at 20–40 DAT. At 41 DAT, newest leaves of the NT and IRO2 lines displayed severe Fe-deficiency symptoms caused by low Fe availability, while RI line 22 grew better than the other lines and exhibited a greater number of tillers (S4 Fig). The SPAD values of NT and IRO2 also increased gradually from 40 DAT, and leaf color recovered, although the recovery was less marked than that of RI lines 21 and 22, and Refre1 lines. At 144 DAT, RI line 22 set more matured grains than did the other transgenic lines and the NT line (Fig 3C).

### Yields of RI lines

**Fig 4:**
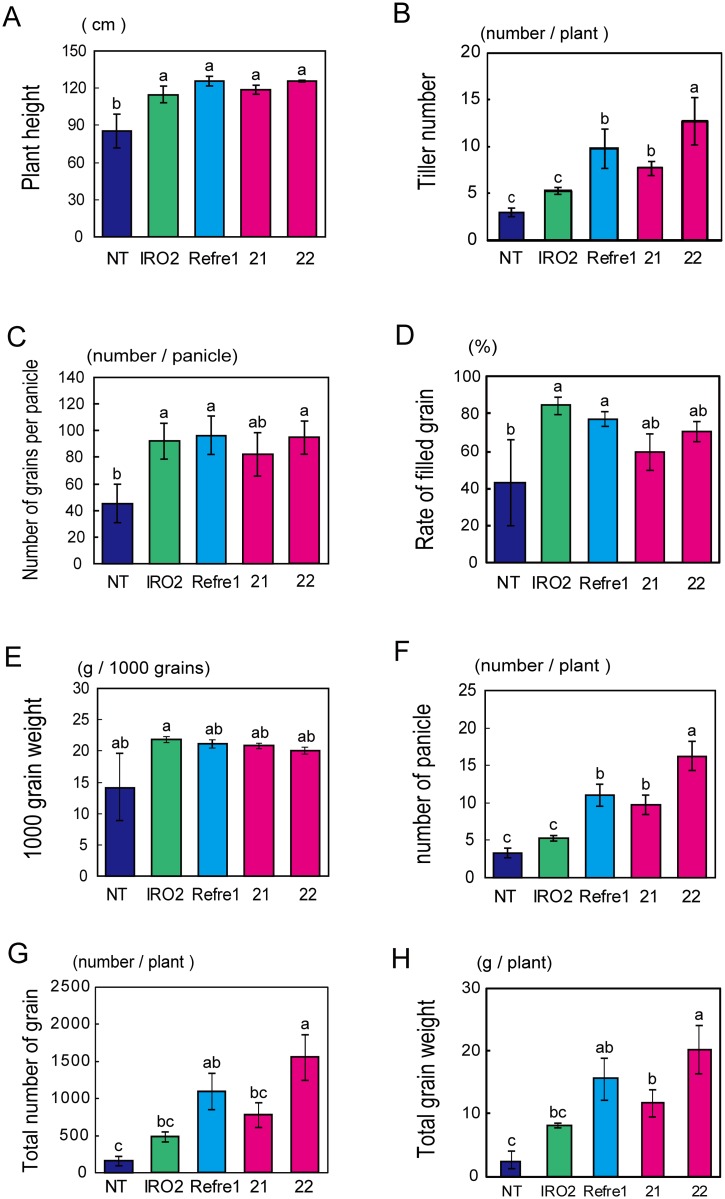
Grain yield and yield components of Tsukinohikari-RI lines cultivated in calcareous soil. (A) Plant height. (B) Tiller number per plant. (C) Number of grains per panicle. (D) Rate of filled grain. (E) 1000-grain weight. (F) Number of panicles per plant. (G) Total number of grains per plant. (H) Total grain weight per plant. Means ± standard error, n = 4. Non-transgenic line (NT), *OsIRO2*-overexpressing transgenic rice (IRO2), transgenic rice possessing *OsIRT1* promoter-*refre1/372*’ (Refre1), and RI lines 21 and 22 (21 and 22) were transplanted to calcareous soil and harvested at 179 DAT. Values with different letters were significantly different by Student’s *t*-test (*p* < 0.05).

We stopped the water supply at 151 DAT, and harvested the plants at 179 DAT. The average plant heights of the IRO2, Refre1, and RI lines were ~120 cm, but that of the NT line was ~90 cm (Fig 4A). The tiller number of RI line 22 was ~4-fold higher than that of the NT line and 2.4-fold higher than that of the IRO2 line (Fig 4B). Both the straw and panicle dry weights (DW) of RI line 22 were ~10-fold higher than that of NT (S5A and S5B Fig). In terms of yield components, the number of grains per panicle, the rate of filled grains, and the 1,000-grain weight of the IRO2 Refre1 line, and RI lines 21 and 22, were similar, and higher than those of the NT line (Fig 4C–4E). The number of grains per panicle of transgenic rice plants was 2-fold higher than that of the NT line (Fig 4C). The rate of filled grains of transgenic plants was 60–80%, while that of the NT line was around 40% (Fig 4D). The 1,000-grain weight of transgenic rice plants tended to be higher than that of the NT line (Fig 4E). Moreover, the number of panicles per plant, the total number of grains, and the total grain weight were higher in RI line 22 than in the NT and OsIRO2 lines (Fig 4F–4H). The total grain weight of RI line 22 was about 9-fold higher than that of the NT line, and 2.5-fold higher than that of the IRO2 line (Fig 4H). NT plants exhibited severely impaired growth, and produced fewer panicles (S6A Fig). The total number of grains and total grain weight were also very low in NT plants (S6B and S6C Fig). In contrast, all transgenic lines, especially RI line 22, produced higher numbers of panicles and total numbers of grains.

### Fe content per plant in RI lines

**Fig 5:**
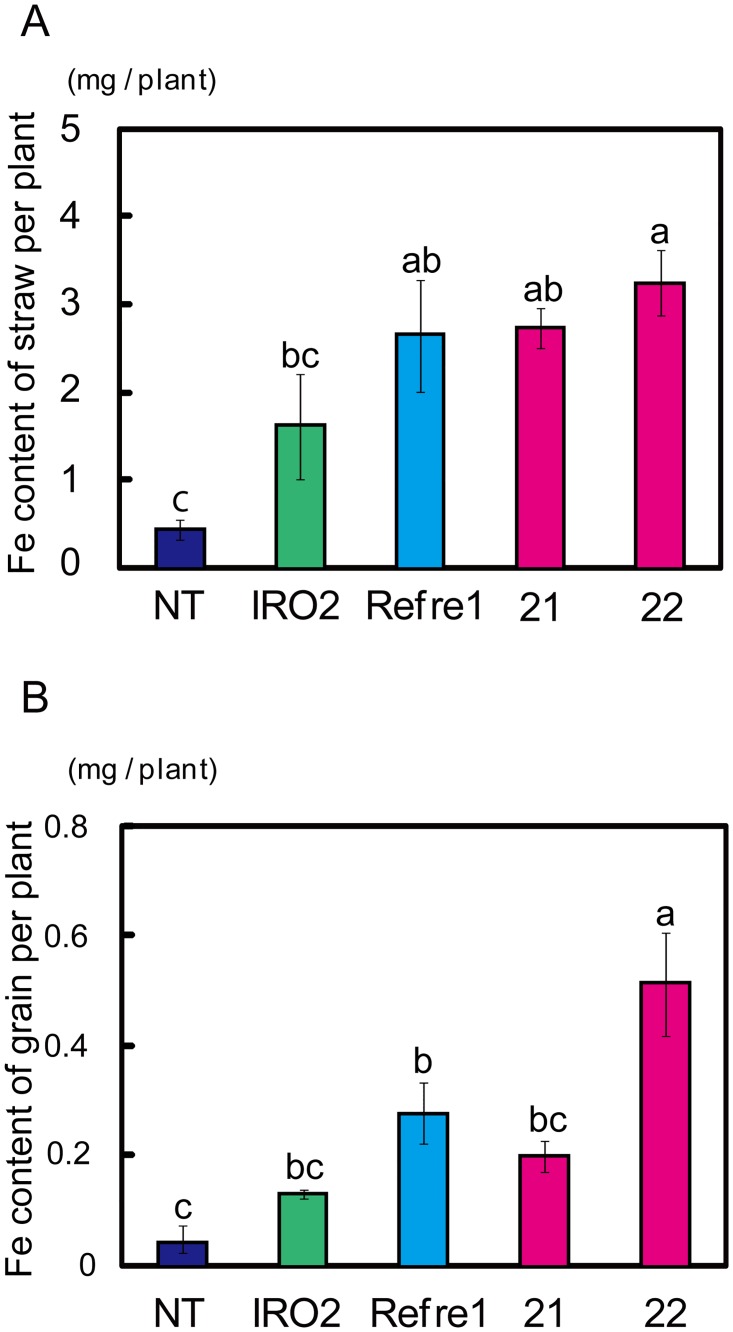
Fe content of Tsukinohikari-RI seeds and straw per plant. (A) Fe content of straw per plant. (B) Fe content of grain (brown rice) per plant. Means ± standard error, n = 4. Values with different letters were significantly different by Student’s *t*-test (*p* < 0.05). 21 and 22, RI transgenic rice lines 21 and 22.

To examine Fe accumulation in RI lines, Fe contents in straw and grain were measured after harvesting. The Fe content in straw of RI line 22 was 3.3 mg/plant, which was 7.3-fold higher than that of the NT line, 2-fold higher than that of the IRO2 line, and 1.2-fold higher than that of the Refre1 line (Fig 5A). Moreover, the Fe content in grain of RI line 22 was around 510 μ g/plant, which was 12-fold higher than that of the NT line, 4-fold higher than that of the IRO2 line, and 2-fold higher than that of the Refre1 line (Fig 5B). Thus, RI line 22 accumulated more Fe in grain and straw than did NT or other transgenic lines.

### Production of Fe-deficiency-tolerant high-biomass rice

The construct harboring the *35S* promoter-*OsIRO2* and *OsIRT1* promoter-*refre1/372* was also introduced into a high-biomass rice cultivar, Tachisugata [33]. Tachisugata-RI lines were produced and T_1_ plants were cultivated in calcareous soil. The shoot lengths and SPAD values of the newest leaves of all cultivated lines were measured at 21 DAT (S7A and S7B Fig). Most of the transgenic lines showed higher heights or SPAD values than regular Tachisugata-NT plants. We selected line 39 as it exhibited the greatest value of plant height multiplied by SPAD value among all transgenic lines (S7C Fig). We confirmed that *OsIRO2* expression in Fe-deficient roots of Tachisugata-RI line 39 was 4-fold higher than in the NT line (data not shown). *Refre1/372* expression was also observed in Fe-deficient roots of line 39 (data not shown).

**Fig 6:**
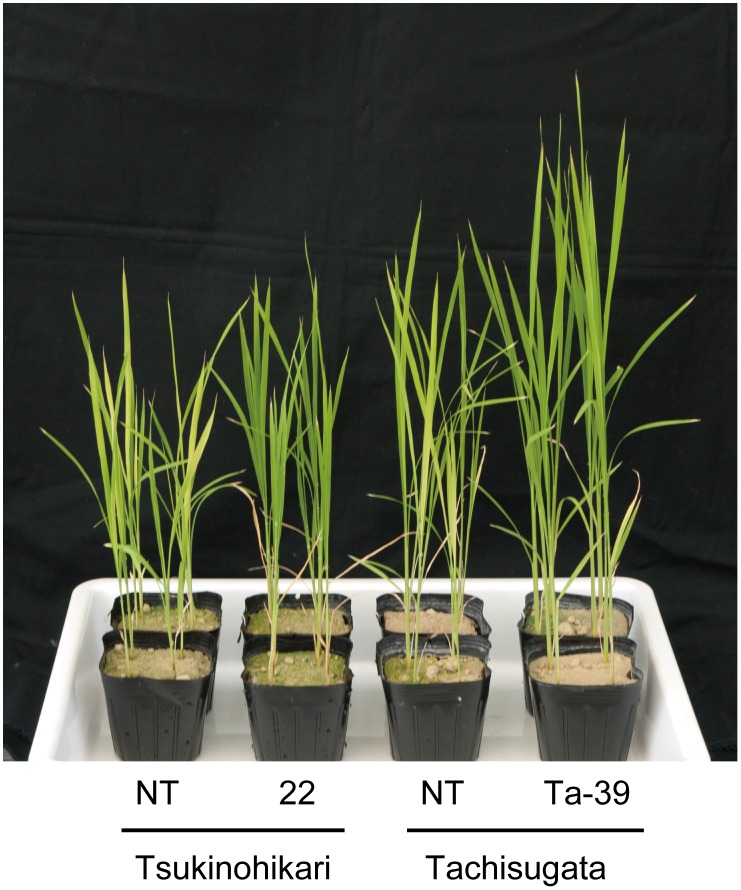
Fe-deficiency-tolerant Tachisugata-RI rice. Photograph taken at 41 DAT to calcareous soil. 22, Tsukinohikari-RI line 22; Ta-39, Tachisugata-RI line 39.

The Tsukinohikari NT line, Tsukinohikari-RI line 22, Tachisugata NT line, and Tachisugata-RI line 39 were cultivated in calcareous soil. Plant heights and SPAD values were measured at 41 DAT. The Tsukinohikari-RI line 22 and the Tachisugata-RI line 39 exhibited better growth, and higher plant heights and SPAD values, compared to the non-transgenic plants (Fig 6 and S8 Fig). Thus, we have successfully produced Fe deficiency-tolerant and high-biomass Tachisugata-RI rice.

## Discussion

### RI rice exhibits enhanced growth in calcareous soil

This study is the first to report a combination of both the reduction and transcription factor strategies to achieve greater tolerance to Fe deficiency than either strategy alone. In calcareous soil, transgenic rice line 22, which harbored both *OsIRO2* and *refre1/372*, grew better than the NT, IRO2, and Refre1 lines, with higher shoot heights and SPAD values (Fig 3 and S4 Fig).

### Role of *OsIRO2* overexpression in Fe-deficiency tolerance

RI lines 21 and 22 and the IRO2 line exhibited higher SPAD values than the NT and Refre1 lines after 10 DAT of Fe deficiency in hydroponic culture (S2A Fig) and within 1 week after transplanting to calcareous soil (Fig 3B). Thus, overexpression of *OsIRO2* was confirmed to improve Fe deficiency tolerance at the early stages of growth. OsIRO2 plays a central role as a regulator of genes involved in strategy II-related Fe uptake and translocation [31–32]. OsIRO2 positively regulates the expression of genes involved in the biosynthesis of NA and DMA, as well as those for DMA and Fe(III)-DMA transporters, under both Fe-deficient and -sufficient conditions, and *OsIRO2* overexpression results in increased DMA secretion under conditions of Fe deficiency [31–32]. We also confirmed higher expression of OsIRO2-downstream genes involved in DMA biosynthesis, such as *OsNAS1*, *OsNAS2*, *OsNAAT1* and *OsDMAS1*, in OsIRO2 lines at an early stage of Fe deficiency (S1 Fig). Thus, the IRO2 line is assumed to be ever ready for strategy II-based tolerance to Fe deficiency before Fe deficiency occurs. Expressions of some of OsIRO2-downstream genes were not higher in RI lines compared to NT, but were higher than Refre1 line (S1 Fig). This might be the effect of enhanced Fe deficiency tolerance conferred by refre1/372.

Nishiyama *et al*. [41] reported the presence of Fe(III)-DMA in rice phloem sap. The abundant DMA in *OsIRO2*-overexpressing lines may contribute not only to Fe uptake but also to Fe translocation within rice plants. In contrast, *refre1/372* was expressed under the control of the *OsIRT1* promoter, which induces gene expression mainly under Fe-deficient conditions in roots of the Refre1 line. Therefore, the Refre1 line likely must encounter Fe-deficient conditions before tolerance can be induced. This might account for the relatively weak tolerance of the Refre1 line to Fe-deficient conditions during the first week of growth in calcareous soil (Fig 3B) and after 10 days of hydroponic culture under Fe-deficient conditions (S2A Fig).

*OsIRO2* expression in the NT line is very low under Fe-sufficient conditions [30]. During 10 days of growth on calcareous soil, *OsIRO2* expression increases gradually in the NT line, and expression of downstream genes, such as *OsNAS1*, *OsNAS2*, *OsNAAT1*, *OsDMAS1*, *TOM1*, and *OsYSL15*, also increases [32]. Due to this induction in NT, the expression levels of these downstream genes become similar in the NT and IRO2 lines after 10 days of growth in calcareous soil [32]. This Fe deficiency-induced function of endogenous *OsIRO2* might account for the relatively small contribution of *OsIRO2* overexpression in the IRO2 compared to the NT line during the mid-stage of growth in calcareous soil (Fig 3A and 3B). Nevertheless, the plant height and SPAD value of RI line 22 were higher than that of the Refre1 line in the mid-stage of growth in calcareous soil (Fig 3A and 3B, S4 Fig), which could have resulted from improved efficiency of Fe availability by enhanced DMA biosynthesis.

The Fe(III)-chelate reductase activity in Tsukinohikari-RI lines 21 and 22 was higher at 3 DAT and decreased at 5 and 7 DAT of Fe-deficient hydroponic culture (S2B Fig). At 5, 7, and 10 DAT, the SPAD values were higher in RI lines 21 and 22 than in the NT and Refre1 lines (S2A Fig). It is assumed that improved Fe nutrition in RI lines 21 and 22 resulted in lower induction of Fe-deficiency signal than in the Refre1 line. Several Fe uptake-related genes in rice, including *OsIRO2* and *OsIRT1*, are positively regulated by transcription factor IDEF1, which is also proposed as a possible Fe sensor in rice cells [38, 42]. Thus, induction of Fe(III)-chelate reductase activity by *OsIRT1* promoter-driven *refre1/372* in RI lines 21 and 22 might not be induced at a high level compared to the Refre1 line at the early Fe-deficient stage because of improved Fe nutrition.

### Role of *refre1/372* expression in Fe-deficiency tolerance

During the middle and late stages of cultivation on calcareous soil, RI lines and the Refre1 line exhibited superior tolerance compared to the NT and IRO2 lines (Fig 3A and 3B). The Refre1 line had a 2-fold greater Fe(III)-chelate reductase activity than did the NT line under Fe-deficient conditions (Fig 2 and S2B Fig). Because of this advantage, Refre1 line may be more resilient than the NT line in the mid- and late stages of growth in calcareous soil. In contrast, the IRO2 line exhibited only slight tolerance during these growth stages. Ogo *et al*. [32] and Ishimaru *et al*. [29] grew the IRO2 and Refre1 lines, respectively, in calcareous soils with low water levels (always less than half of the pot height), which led to significant tolerance compared to the NT line [29, 32, personal communications]. The calcareous soil test of the present study featured a higher water level (continuous submergence). Diffusion of MAs might be increased under such conditions, possibly accounting for the relatively weak contribution of *OsIRO2* overexpression. Araki *et al*. [43] reported dose-dependent effects of DMA application to hydroponic culture solution on improvement of Fe nutrition. Thus, enhancement of the reduction strategy mediated by *refre1/372* would be particularly effective in supporting Fe uptake when strategy II-based uptake is insufficient during the mid- and late stages of submerged growth.

In summary, RI lines 21 and 22 were tolerant to Fe deficiency during the early and mid-late stages of cultivation in calcareous soil because of a combination of *OsIRT1* promoter-induced *refre1/372* expression and overexpression of *OsIRO2*.

### Higher plant survival rate and increased tiller number were the main factors improving grain yield in RI lines

The total grain weight of RI line 22 was 9-fold higher than that of the NT line (Fig 4H). All four plants of the IRO2, Refre1, and RI transgenic lines developed seeds and attained a respectable total grain weight during calcareous soil cultivation (Fig 3C, S6B and S6C Fig). In contrast, in the NT line, the total number of grains and total grain weight were low in two of the four plants and one plant had no filled grains (S6B and S6C Fig). NT plants did not survive well in calcareous soil until the end of their life cycle. The number of surviving plants per unit area in calcareous soil is key to achieve high yields.

The number of grains per panicle, the rate of filled grains, and the 1,000-grain weight were similar among the transgenic lines (Fig 4C–4E). In contrast, the tiller number and number of panicles were higher in RI line 22 than in the other transgenic lines (Fig 4B and 4F). The total number of grains and the total grain weight were also higher in RI line 22 (Fig 4G and 4H). This suggests that tiller number and the number of panicles might affect grain yields in calcareous soil. The number of panicles is determined by the tiller number, and reflected Fe-deficiency-tolerance during early growth. In fact, RI line 22 exhibited a higher plant height and SPAD value during the early and mid-stages of growth in calcareous soil (Fig 3A, 3B and S4 Fig). Therefore, it is crucial for rice cultivated on calcareous soil to survive the early stage of growth. The panicle initiation date was 3 or 4 days earlier in line 22 than in the other transgenic lines, and 1 week earlier than in the NT line (data not shown). Such early panicle initiation may also have contributed to the higher yield of line 22.

### Enhanced Fe uptake and translocation to grains in RI lines

The per-plant Fe contents of straw and grain were markedly higher in RI lines compared to the NT line (Fig 5A and 5B). This was likely due to a combination of enhanced Fe uptake ability from soil by *refre1/372* induction and *OsIRO2* overexpression, and enhanced Fe translocation mediated by increased NA and DMA levels caused by *OsIRO2* overexpression. Masuda *et al*. [39] and Lee *et al*. [44] showed that enhancement of NA and DMA productivity enhances Fe translocation in rice plants, yielding grain of high Fe content. Masuda *et al*. [45] also showed that MA production increased the Fe content of rice grains. In the present calcareous test, the mean Fe concentrations in brown seeds were 20, 18, 19, 19, and 30 μg/g in the NT, IRO2, Refre1, RI 21, and RI 22 lines, respectively; these levels did not differ significantly (data not shown). Takahashi *et al*. [46] showed that NA-chelated metals, such as Fe and Zn, are essential, especially during grain maturation. Rice plants may have a system that mediates accumulation of a certain amount of Fe in each grain. Under Fe-limited conditions, NT plants may set fewer seeds to ensure distribution of the limited Fe available to all seeds. Therefore, NT plants have many unfilled grains (Fig 4D). In contrast, transgenic lines with improved Fe uptake and translocation likely produce and distribute Fe to, a greater number of grains, but accumulate only the required amount of Fe in each seed. Ogo *et al*. [32] reported higher Fe concentration in brown seeds of the IRO2 line (up to 20 μg/g) than that of the NT line (6 μg/g) which is lower than that in ordinary grains of this cultivar (Tsukinohikari). This might account for the 3-fold higher Fe concentration in grains of the IRO2 line by Ogo *et al*. [32].

Irrespective of the similar Fe concentrations in each grain of the NT, IRO2, Refre1 and RI lines in the present report, the markedly increased grain weight in the IRO2, Refre1, and RI lines resulted in higher Fe accumulation in grains per plant (Figs 4H and 5B). Enhanced Fe(III)-reduction and transcription factor strategies may thus be used for Fe biofortification of rice seeds cultivated under low Fe conditions. On the other hand, other strategies are also required in combination to consistently accumulate more Fe in seeds [47].

### Production of an Fe-deficiency-tolerant, high-biomass rice line

We successfully produced an Fe-deficiency-tolerant high-biomass Tachisugata rice cultivar (Fig 6, S7 and S8 Figs). This cultivar has long and thick culms, is highly resistant to lodging, and is adapted to direct-sowing cultivation. Thus, this cultivar is suitable for production of biomass or whole-crop silage for use as fodder [33]. Moreover, an efficient on-site ethanol production system using Tachisugata rice is under development [48]. Therefore, the Fe deficiency-tolerant Tachisugata rice produced in this study may be useful. Moreover, Fe deficiency-tolerant lines of other high-biomass crops—such as maize, sugarcane, or sorghum—could be produced using Fe(III)-chelate reductase genes and enhancement of the expression of Fe homeostasis-related transcription factors, such as *OsIRO2* homologs.

### Other approaches to production of Fe-deficiency-tolerant rice lines

Transgenic rice plants harboring *IDEF1* under the control of the barley Fe deficiency-inducible *IDS2* promoter exhibited improved Fe-deficiency tolerance during early growth, but not during the mid-late stages in calcareous soils [42, 49]. Ishimaru *et al*. [50] identified the rice phenolic efflux transporter, PEZ1, and reported that *PEZ1*-overexpressing rice showed enhanced growth in calcareous soil. Kobayashi *et al*. [49] reported that the knock-down of Fe-binding hemerythrin RING ubiquitin ligases, *OsHRZ1* and *OsHRZ2*, in rice resulted in enhanced expression of Fe deficiency-inducible genes involved in Fe utilization, and enhanced tolerance to Fe deficiency during growth in calcareous soil. This *HRZ*-knockdown rice also showed markedly higher accumulation of Fe in grains and shoots under both Fe-sufficient and -deficient conditions [49], and thus is a promising candidate for both improved production in problem soils and biofortification. Introduction of barley MA biosynthesis genes (*HvNAS1*, *HvNAAT-A*,*B*, or *IDS3*) also rendered rice plants tolerant to Fe deficiency [26,27,51]. These reports suggest that rice lines with enhanced tolerance to Fe deficiency could be produced using a combination of these various transgenic approaches.

### Conclusion

RI rice lines had higher yields than the NT line because of the enhanced tolerance to low Fe availability at both the early and mid-late stages of growth in calcareous soil. All plants survived and tiller number increased at all growth stages. Also, Fe was effectively transported to seeds. Thus, the rate of filled grains improved at the grain-maturation stage. Therefore, we have successfully produced transgenic rice lines affording increased grain yields in calcareous soils by means of both enhanced reduction (Strategy I) and chelation (Strategy II). We also successfully produced a high-biomass rice variety with increased tolerance to Fe deficiency for increased grain yield and higher biomass productivity.

## Supporting information

S1 Fig Gene expression levels of *OsIRO2*, *refre1/372*, and representative OsIRO2-downstream genes. Non-transgenic line (NT), and transgenic rice expressing the *35S* promoter-*OsIRO2* (IRO2), the *OsIRT1* promoter-*refre1/372*’ (Refre1), and the RI lines (21 and 22) were grown in hydroponic culture solution for 10 days and transferred to Fe-deficient culture solution for 1 day. Total RNA was extracted from roots and gene expression levels were analyzed by quantitative RT-PCR. Data are shown as copies of each gene / *OsTublin1* copies [means ± standard error of technical replication, n = 3 except for *OsNAAT1* (n = 1)]. n.d., not detected.
(PDF)

S2 Fig Relative chlorophyll content and Fe(III)-chelate reductase activity during hydroponic growth. (A) SPAD value of the newest leaves. (B) Fe(III)-chelate reductase activity. Non-transgenic line (NT), and transgenic rice expressing the *35S* promoter-*OsIRO2* (IRO2), the *OsIRT1* promoter-*refre1/372*’ (Refre1), and the RI lines (21 and 22) were grown in hydroponic culture solution for 10 days and then transferred to hydroponic culture solution without Fe. SPAD values and Fe(III)-chelate reductase activities of roots were measured at the indicated days after onset of Fe-deficiency treatment (means ± standard error, n = 4).
(JPG)

S3 Fig Photograph of T_2_ plants growing in calcareous soil in a greenhouse on the day of transplantation. (JPG)

S4 Fig Photograph of plants at 41 DAT to calcareous soil. NT, non-transformant; IRO2, *OsIRO2*-overexpressing transgenic rice; Refre1, *OsIRT1* promoter-*refre1/372*’ transgenic rice; 21 and 22, RI transgenic rice lines 21 and 22.
(JPG)

S5 Fig Total weight of straw and panicle of rice lines cultivated in calcareous soil. (A) Dry weight of straw per plant. (B) Dry weight of panicle per plant. Plants were harvested after 179 days of growth in calcareous soil, and the straw and panicle weight per plant was analyzed (means ± standard error, n = 4). Values followed by different letters were significantly different according by Tukey–Kramer’s HSD test (*p* < 0.05). NT, non-transformant; IRO2, *OsIRO2*-overexpressing transgenic rice; Refre1, *OsIRT1* promoter-*refre1/372*’ transgenic rice; 21 and 22, RI lines 21 and 22.
(JPG)

S6 Fig Number of panicles and grains and total grain weight per plant of four independent plants. (A) Number of panicles per plant. (B) Total number of grains per plant. (C) Total grain weight per plant. Each bar shows the value of a single plant (T_2_ sub-lines of transgenic plants or NT plants) cultivated in calcareous soil. NT, non-transformant; IRO2, *OsIRO2*-overexpressing transgenic rice; Refre1, *OsIRT1* promoter-*refre1/372*’ transgenic rice; 21 and 22, RI lines 21 and 22.
(JPG)

S7 Fig Selection of Tachisugata-RI lines cultivated in calcareous soil. (A) Plant height of individual lines. (B) SPAD value of the newest leaves of individual lines. (C) Value of plant height multiplied by the SPAD value of each plant used for line selection. Plant heights and SPAD values were measured at 21 DAT to calcareous soil. We selected Tachisugata-RI line 39 as it yielded the highest value of (C). A–E on the X-axis indicate non-transgenic Tachisugata plants. Numbers on the X-axis represent individual Tachisugata-RI lines. White circles on each line are data from individual plants. Red circles on the NT lines are the mean values of eight NT plants. Black circles are the mean values of eight plants of each Tachisugata-RI line. The red dotted line shows the mean values of all 40 NT plants.
(JPG)

S8 Fig Growth test of Tsukinohikari-RI and Tachisugata-RI transgenic rice lines on calcareous soil. (A) Height of individual plants. (B) SPAD value of individual plants. TK-NT, Tsukinohikari non-transgenic plants; 22, Tsukinohikari-RI line 22; Ta-NT, Tachisugata non-transgenic plants; Ta-39, Tachisugata-RI line 39. Values for eight individual plants of each non-transgenic and transgenic line are shown in individual bars.
(JPG)
