# Supplementary figures and images for: A new transgenic rice line exhibiting enhanced ferric iron reduction and phytosiderophore production confers tolerance to low iron availability in calcareous soil
Source: PLoS One. 2017 Mar 9;12(3):e0173441. doi: 10.1371/journal.pone.0173441 (PMC5344405; doi:10.1371/journal.pone.0173441)

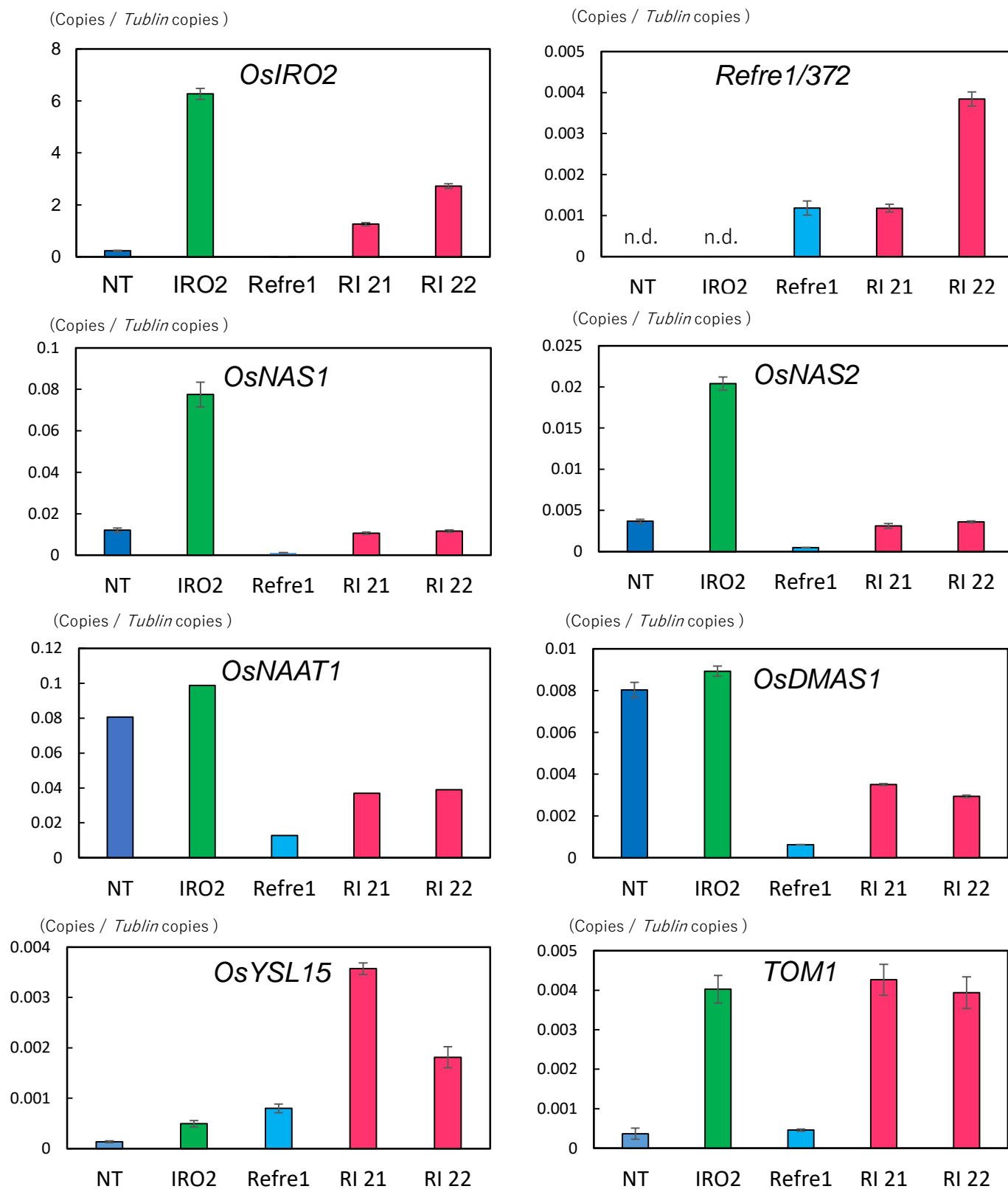

Figure S1

Supplement: S1 Fig — Non-transgenic line (NT), and transgenic rice expressing the 35S promoter-OsIRO2 (IRO2), the OsIRT1 promoter-refre1/372’ (Refre1), and the RI lines (21 and 22) were grown in hydroponic culture solution for 10 days and transferred to Fe-deficient culture solution for 1 day. Total RNA was extracted from roots and gene expression levels were analyzed by quantitative RT-PCR. Data are shown as copies of each gene / OsTublin1 copies [means ± standard error of technical replication, n = 3 except for OsNAAT1 (n = 1)]. n.d., not detected. (PDF) [file pone.0173441.s001.pdf]

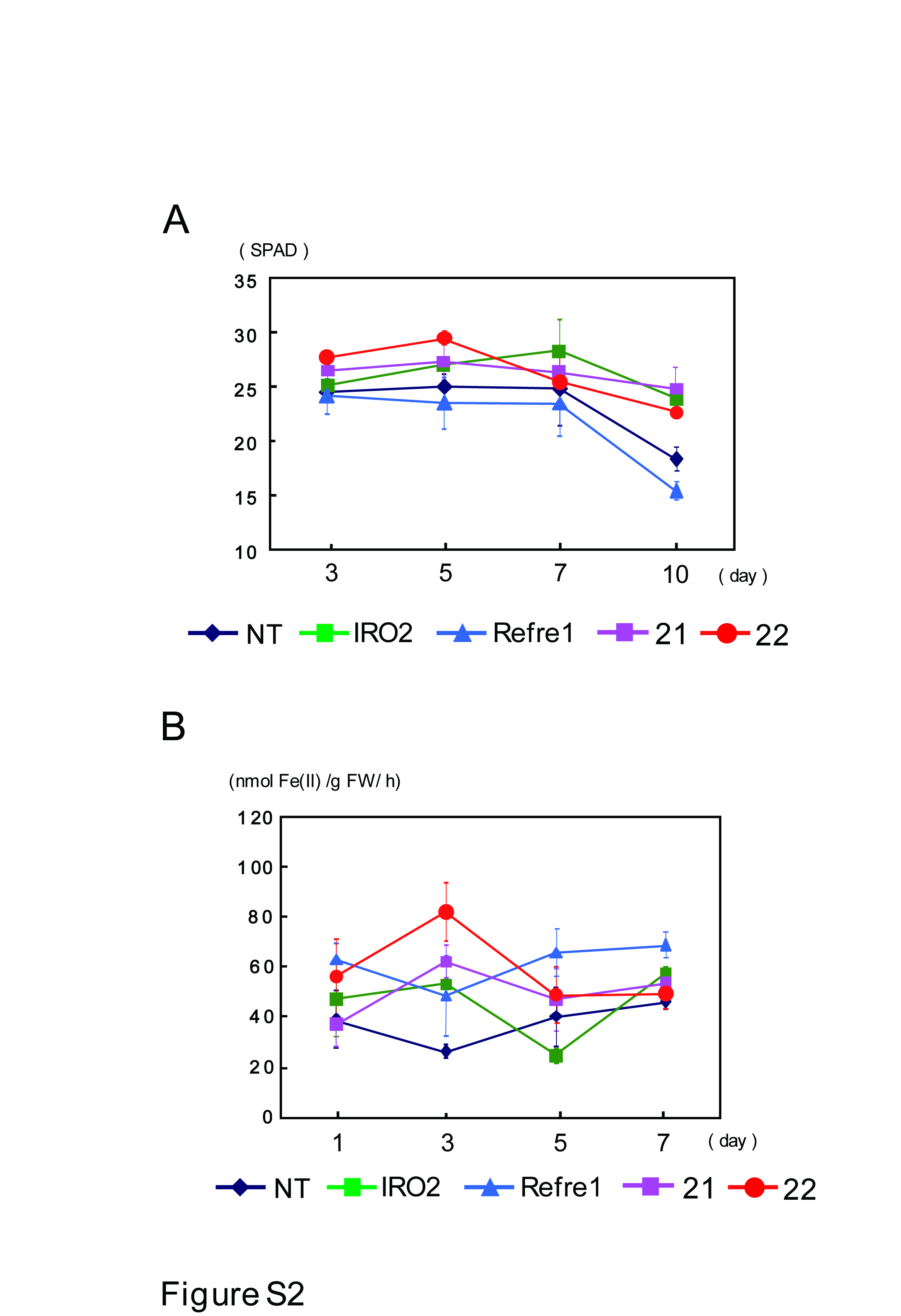

Supplement: S2 Fig — (A) SPAD value of the newest leaves. (B) Fe(III)-chelate reductase activity. Non-transgenic line (NT), and transgenic rice expressing the 35S promoter-OsIRO2 (IRO2), the OsIRT1 promoter-refre1/372’ (Refre1), and the RI lines (21 and 22) were grown in hydroponic culture solution for 10 days and then transferred to hydroponic culture solution without Fe. SPAD values and Fe(III)-chelate reductase activities of roots were measured at the indicated days after onset of Fe-deficiency treatment (means ± standard error, n = 4). (JPG) [file pone.0173441.s002.jpg]

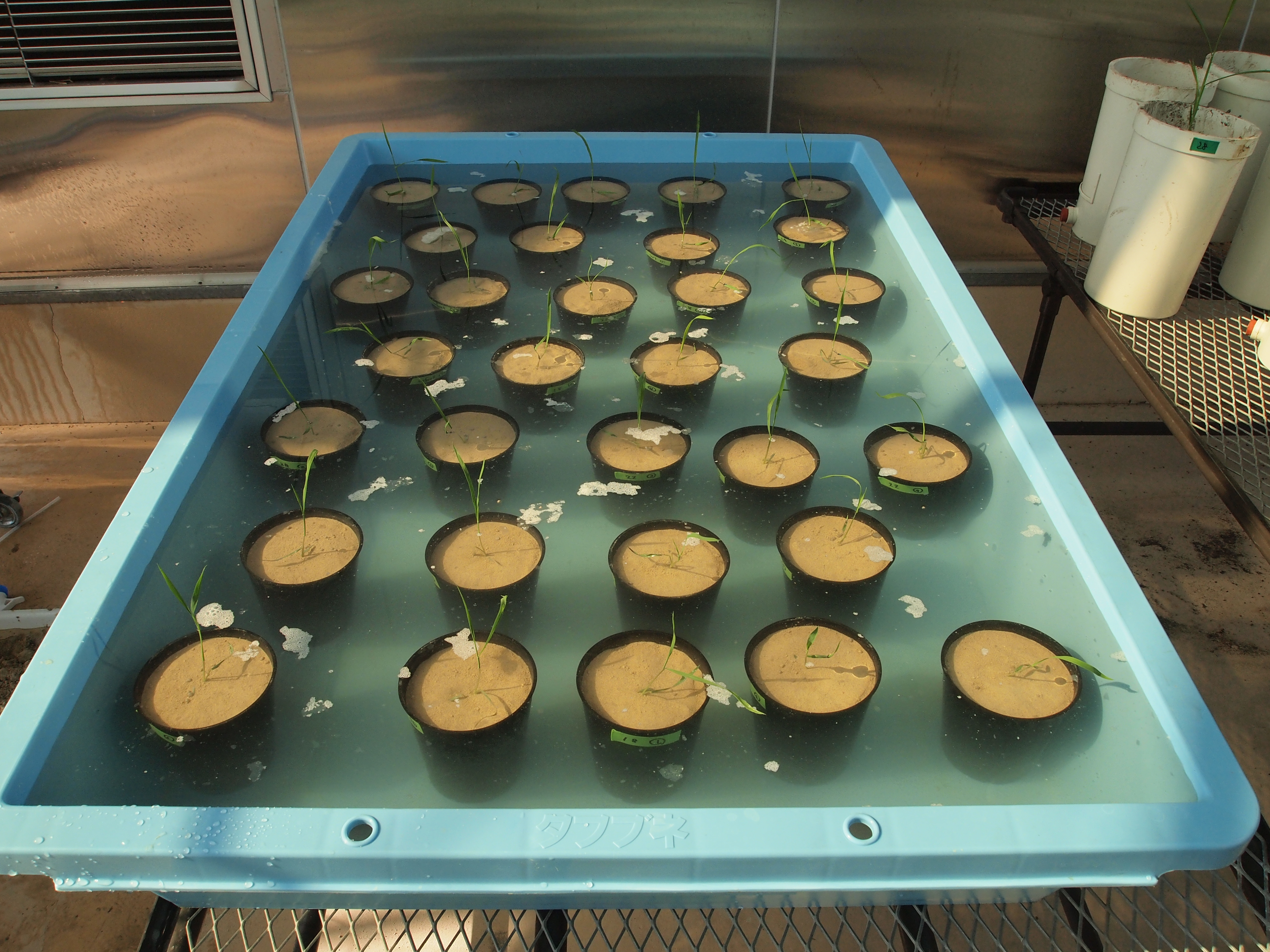

Supplement: S3 Fig — (JPG) [file pone.0173441.s003.JPG]

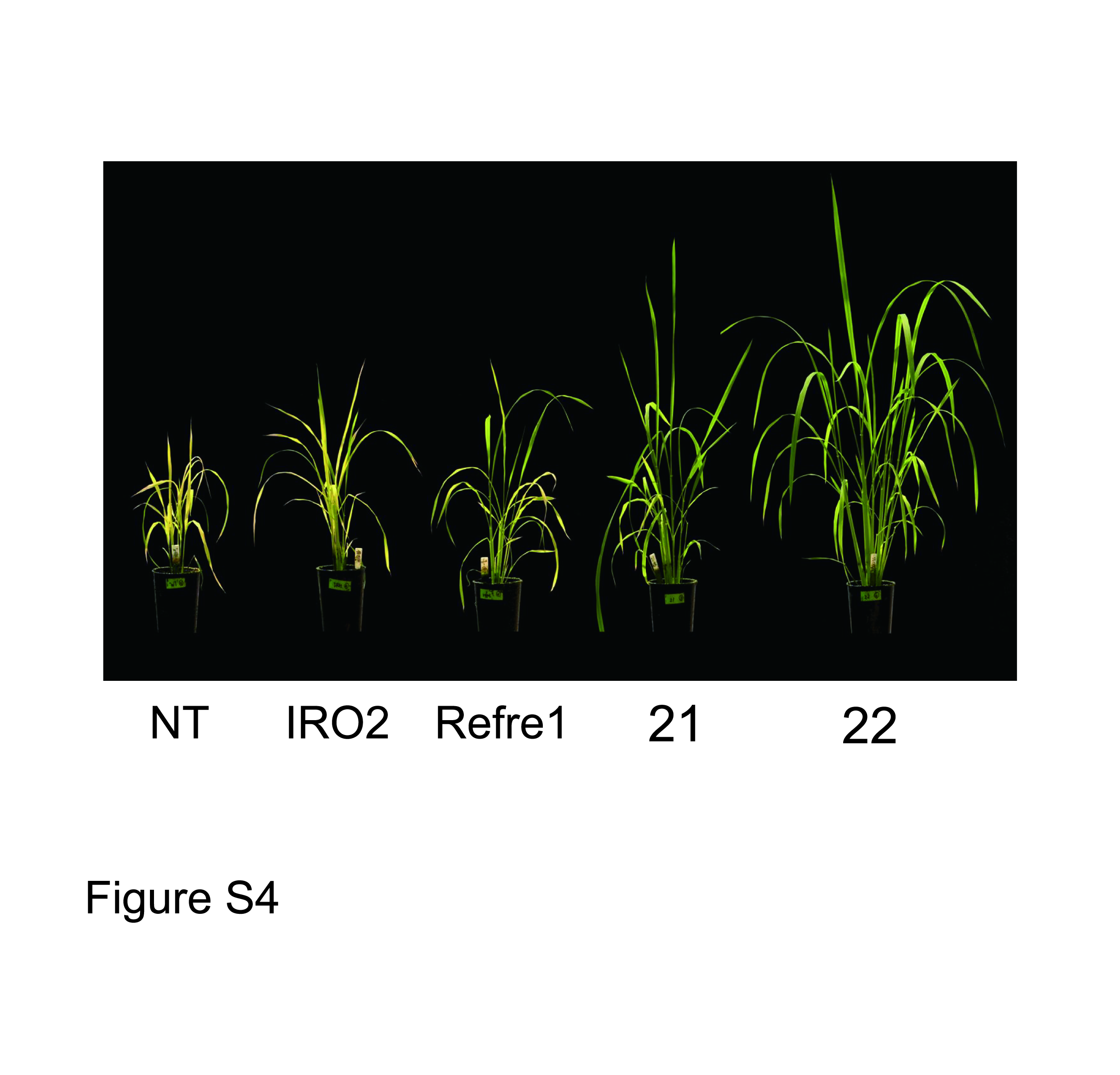

Supplement: S4 Fig — NT, non-transformant; IRO2, OsIRO2-overexpressing transgenic rice; Refre1, OsIRT1 promoter-refre1/372’ transgenic rice; 21 and 22, RI transgenic rice lines 21 and 22. (JPG) [file pone.0173441.s004.jpg]

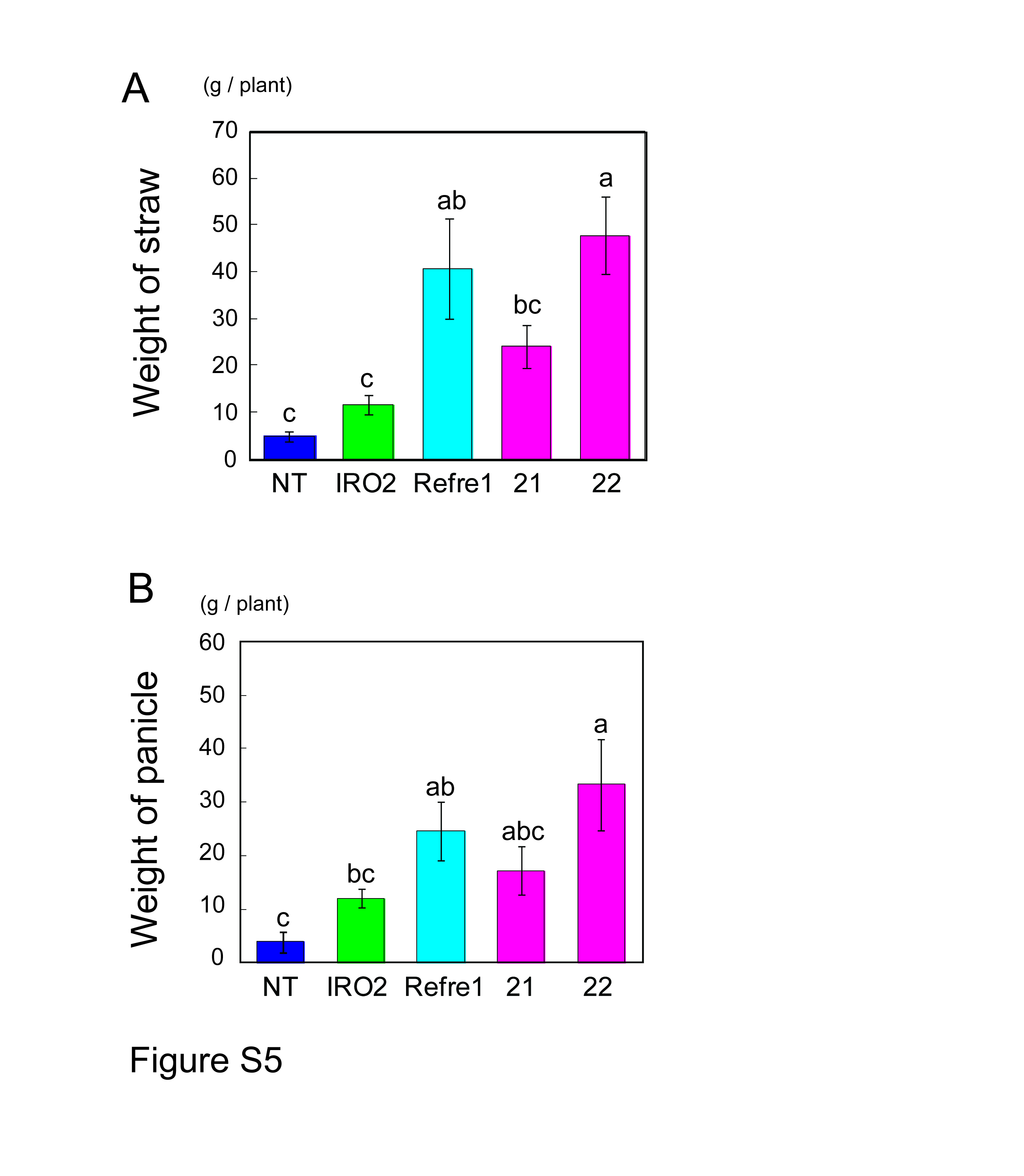

Supplement: S5 Fig — (A) Dry weight of straw per plant. (B) Dry weight of panicle per plant. Plants were harvested after 179 days of growth in calcareous soil, and the straw and panicle weight per plant was analyzed (means ± standard error, n = 4). Values followed by different letters were significantly different according by Tukey–Kramer’s HSD test (p < 0.05). NT, non-transformant; IRO2, OsIRO2-overexpressing transgenic rice; Refre1, OsIRT1 promoter-refre1/372’ transgenic rice; 21 and 22, RI lines 21 and 22. (JPG) [file pone.0173441.s005.jpg]

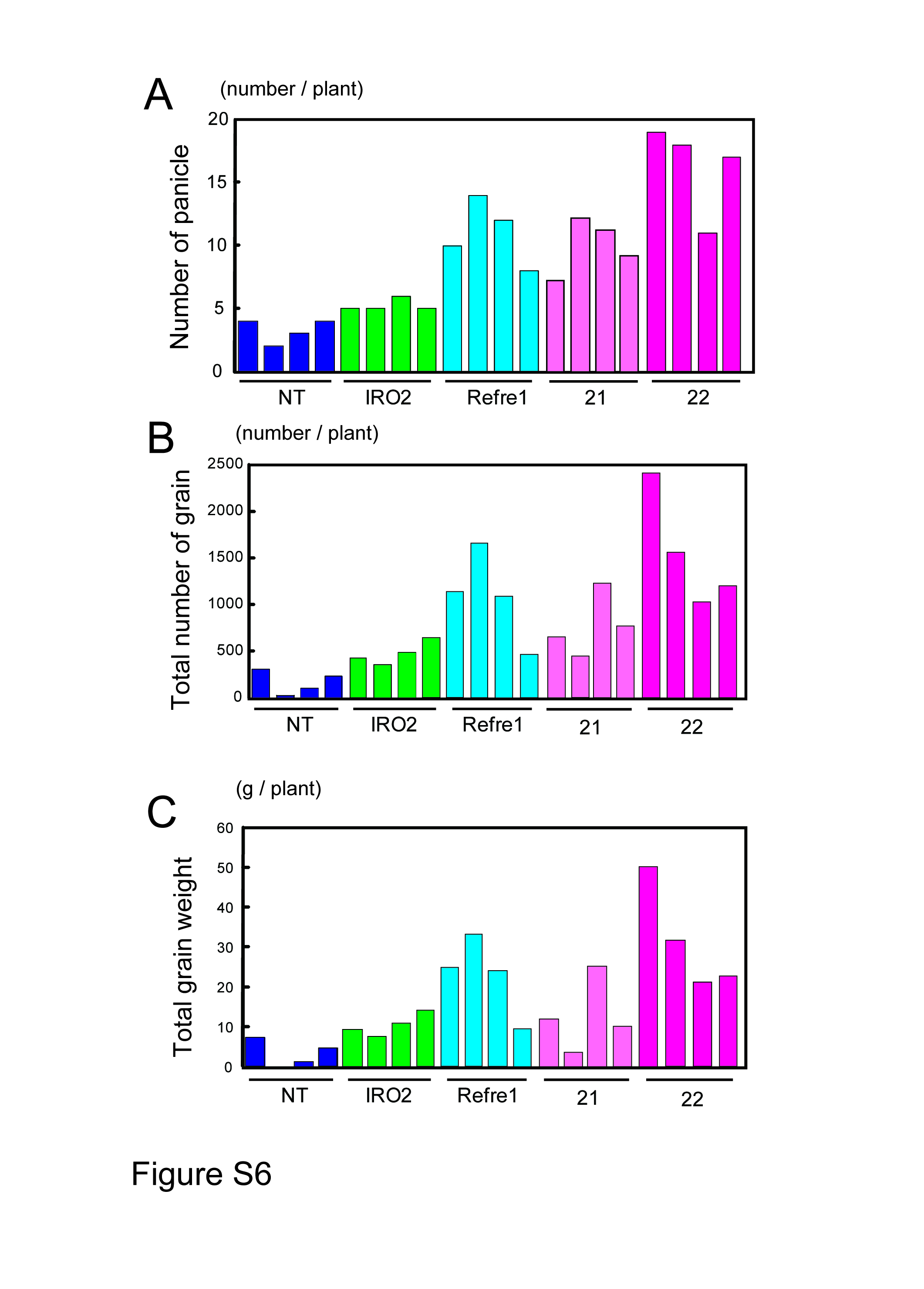

Supplement: S6 Fig — (A) Number of panicles per plant. (B) Total number of grains per plant. (C) Total grain weight per plant. Each bar shows the value of a single plant (T2 sub-lines of transgenic plants or NT plants) cultivated in calcareous soil. NT, non-transformant; IRO2, OsIRO2-overexpressing transgenic rice; Refre1, OsIRT1 promoter-refre1/372’ transgenic rice; 21 and 22, RI lines 21 and 22. (JPG) [file pone.0173441.s006.jpg]

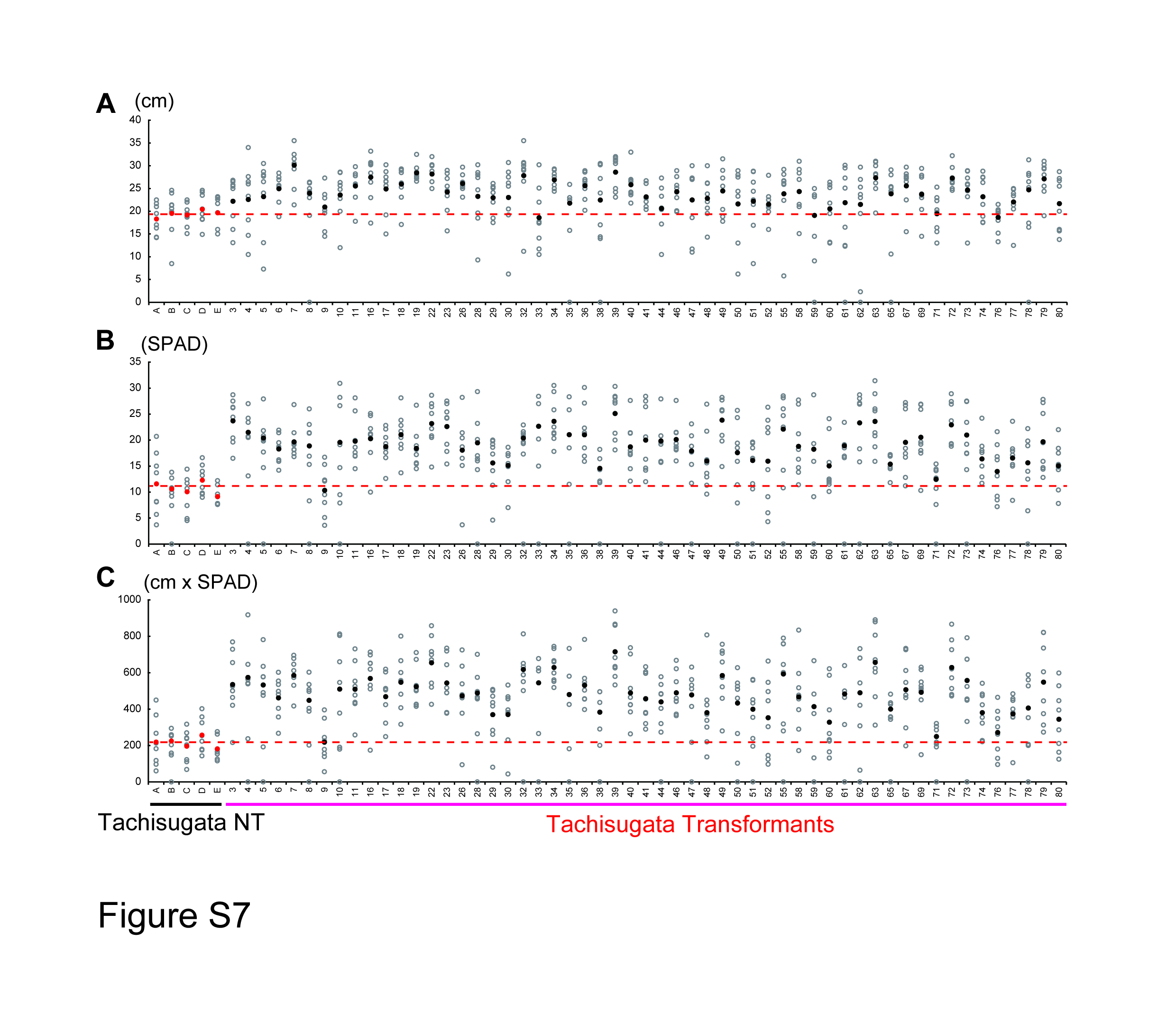

Supplement: S7 Fig — (A) Plant height of individual lines. (B) SPAD value of the newest leaves of individual lines. (C) Value of plant height multiplied by the SPAD value of each plant used for line selection. Plant heights and SPAD values were measured at 21 DAT to calcareous soil. We selected Tachisugata-RI line 39 as it yielded the highest value of (C). A–E on the X-axis indicate non-transgenic Tachisugata plants. Numbers on the X-axis represent individual Tachisugata-RI lines. White circles on each line are data from individual plants. Red circles on the NT lines are the mean values of eight NT plants. Black circles are the mean values of eight plants of each Tachisugata-RI line. The red dotted line shows the mean values of all 40 NT plants. (JPG) [file pone.0173441.s007.jpg]

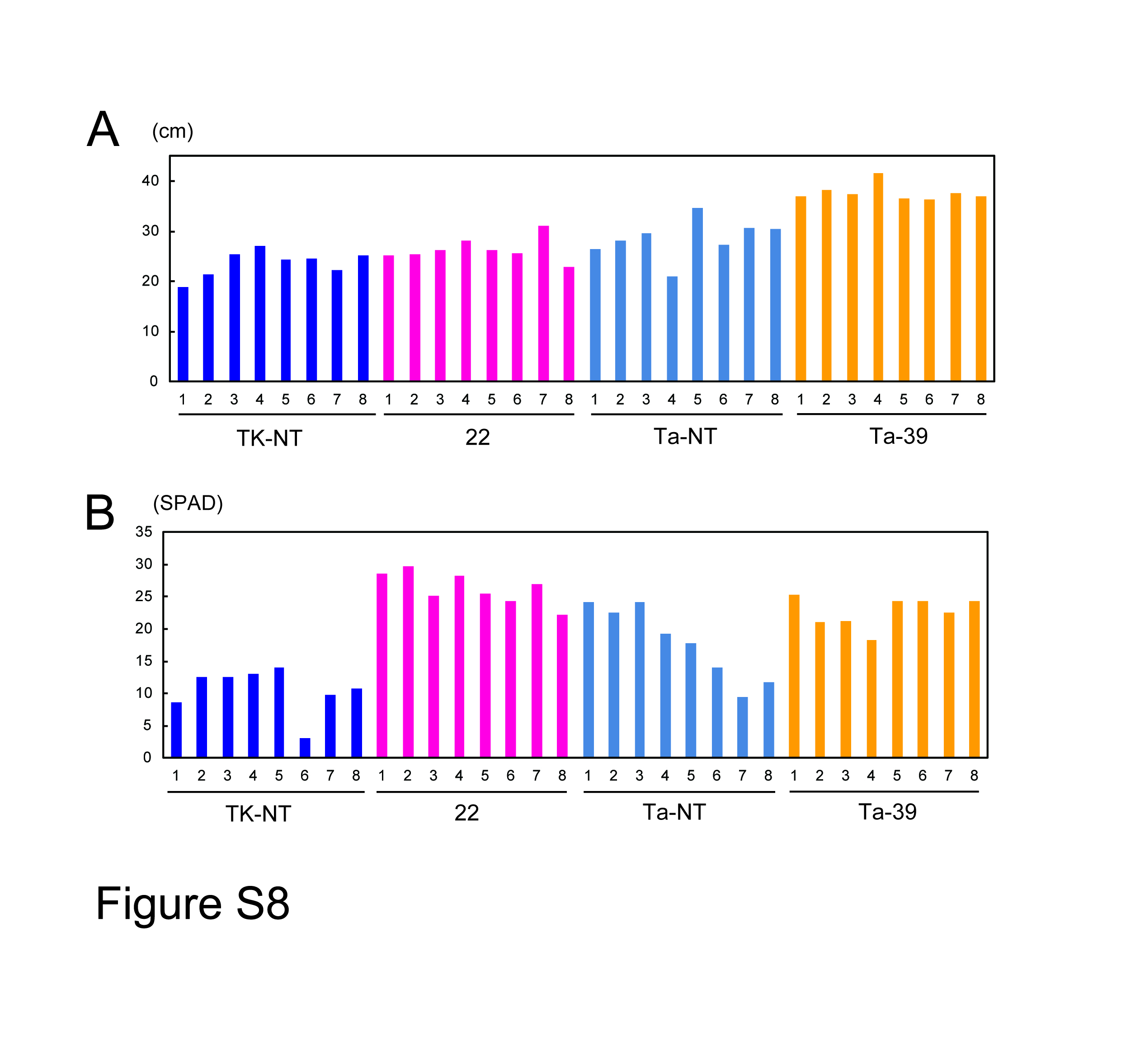

Supplement: S8 Fig — (A) Height of individual plants. (B) SPAD value of individual plants. TK-NT, Tsukinohikari non-transgenic plants; 22, Tsukinohikari-RI line 22; Ta-NT, Tachisugata non-transgenic plants; Ta-39, Tachisugata-RI line 39. Values for eight individual plants of each non-transgenic and transgenic line are shown in individual bars. (JPG) [file pone.0173441.s008.jpg]
